# Perceptions and experiences of fertility preservation in female patients with cancer in Greece

**DOI:** 10.1186/s12905-024-02955-x

**Published:** 2024-02-09

**Authors:** Anna-Maria Taniskidou, Polychronis Voultsos, Vasileios Tarlatzis, Eleni Timotheadou

**Affiliations:** 1 Laboratory of Forensic Medicine & Toxicology (Division: Medical Law and Ethics), School of Medicine, Faculty of Health Sciences, Aristotle University of Thessaloniki, University Campus, Thessaloniki, GR 54124 Greece; 2 1st Department of Obstetrics and Gynaecology, School of Medicine, Faculty of Health Sciences, Aristotle University of Thessaloniki, University Campus, Thessaloniki, GR 54124 Greece; 3 Department of Medical Oncology, School of Medicine, Faculty of Health Sciences, Papageorgiou Hospital, Aristotle University of Thessaloniki, Thessaloniki, Greece

**Keywords:** Fertility preservation, Oncofertility, Women with cancer, Biological Parenthood

## Abstract

**Background:**

As advances in oncology have led to remarkable and steady improvements in the survival rates of patients with cancer and anticancer treatment can cause premature ovarian failure in women, fertility preservation (FP) has become a global public health concern and an integral part of the care for women diagnosed with cancer during reproductive age. However, for various reasons, FP remains underutilized for patients with cancer. There are substantial gaps in our knowledge about women’s experiences and perceptions of the issue. This study aims to contribute to bridging that gap.

**Methods:**

This prospective qualitative study was conducted from March 2018 to February 2023. A combination of purposive and snowball sampling was used. Data were collected by semistructured interviews with nineteen reproductive-age women who had been recently diagnosed with cancer. Data were classified and analysed with a thematic analysis approach.

**Results:**

A variety of distinct themes and subthemes emerged from the analysis of the interview data. The cancer diagnosis emerged as a factor that considerably affects the women’s attitudes towards biological parenthood: It can further increase their (strong) previous desire or decrease their previous (weak) desire. Women with a recent cancer diagnosis had not received adequate and multidisciplinary counselling, including clear and sufficient information. However, participants felt satisfied with the information they received because they either received the information they requested or remained in denial about the need to be informed (i.e., because they felt overwhelmed after the cancer diagnosis). Embryo cryopreservation emerged as a less desirable FP option for women with cancer. Participants showed respect for human embryos, not always for religious reasons. Surrogacy emerged as the last resort for most participants. Religious, social or financial factors did play a secondary (if any) role in women’s decision-making about FP. Finally, male partners’ opinions played a secondary role in most participants’ decision-making about FP. If embryo cryopreservation was the selected option, partners would have a say because they were contributing their genetic material.

**Conclusions:**

The findings that emerged from the data analysis were partly consistent with prior studies. However, we identified some interesting nuances that are of clinical importance. The results of this study may serve as a starting point for future research.

**Supplementary Information:**

The online version contains supplementary material available at 10.1186/s12905-024-02955-x.

## Introduction

As advances in oncology in recent decades have led to high peak improvements in the survivorship rates of young people diagnosed with cancer, fertility preservation (FP) has become an important consideration for patients with cancer [1–3]. Female patients of reproductive age undergoing gonadotoxic anticancer therapy are at high risk for premature ovarian failure (POF) and infertility or subfertility [3]. Chemotherapy, radiotherapy or their combination are a great threat to fertility among women with cancer. FP has become an integral part of the care of young female cancer patients (FCPs). It is increasingly recognized that these patients, especially those undergoing novel oncological treatments, have to deal with their quality of life (QoL) in addition to a life-threatening diagnosis [1, 4–7]. Young FCPs have to make difficult decisions involving the concept of QoL, which is a vague and complex concept with multiple definitions and ‘many diverse facets and components’ [4].

It is widely argued in the literature that reproductive capacity in humans and especially in women is strictly related to their (reproductive) autonomy and well-being [6–8]. FP should be regarded as a medical treatment, especially in light of the new holistic-positive definition of the concept of health. Indeed, there is a need for a ‘shift from a biomedical approach of cancer treatment towards a holistic understanding of the impact of cancer on the individual’s quality of life’ [7]. Oncofertility, namely, FP for cancer patients, is a novel discipline [9]. Oncofertility involves medical, surgical and laboratory procedures to preserve fertility in young FCPs whose reproductive potential is at high risk of being lost [2, 7, 10, 11]. Most importantly, oncofertility involves various health care professionals (HCPs) and many difficult ethical dilemmas.

There are various FP methods that are currently available to a young female patient recently diagnosed with cancer. Oocyte or embryo cryopreservation are well-established methods for adults and postpubertal girls, and ovarian tissue cryopreservation is being offered as an experimental method for prepubertal girls and adult female patients whose medical conditions do not allow for their cancer treatment to be postponed for at least two weeks [2].

While FP is of great importance to reproductive-aged FCPs, it remains underutilized in clinical practice for various reasons. Most importantly, it is well known in the literature that reproductive-aged FCPs are often provided with inadequate information about the FP options that are available to them. While the literature states that HCPs should always provide optimal counselling [1], many primary care physicians are unaware of the possible negative impact of anticancer treatment on patient fertility [12]. Health care professionals’ lack of knowledge about how to manage FP conversations with young FCPs seems to be a major reason behind these patients’ unmet needs regarding FP [13]. Early referral to specialists who are able and willing to discuss FP options is strongly recommended [12].

Ultimately and most importantly, it should be pointed out that very substantial knowledge gaps still remain in the available literature regarding cancer patients’ experiences, and they need to be filled [14, 15]. More specifically, substantial knowledge gaps have been identified regarding cancer patients’ specific feelings or needs for FP options [14, 16]. This study aimed to contribute to filling these knowledge gaps.

## Study design

The present work was a prospective qualitative research study based on in-depth interviews conducted with FCPs of reproductive age with a recent diagnosis of cancer who are considering or have considered FP as a means of overcoming the risk of cancer treatment-induced infertility. This qualitative descriptive study was conducted from March 2018 to February 2023. Participant recruitment and data collection took a long time because of the COVID-19 pandemic. Thematic analysis was used as the methodological orientation of the study.

### Inclusion criteria

Τhis study included (a) women of reproductive age with a diagnosis of primary cancer who (b) were able and willing to reproduce and could complete interviews prior to or during chemotherapy treatment.

### Exclusion criteria

Women who (a) were not able to have FP discussions at the time of the interview and (b) women with bilateral ovarian cancer were excluded from the sample of this study.

## Materials and methods

In-depth individual semistructured face-to-face interviews were conducted after approval through the Ethics and Deontology Committee of the Medical School of the Aristotle University of Thessaloniki (Reference number: 3.663/2.5.2018) and the Scientific Committee of the ‘Papageorgiou’ Hospital of Thessaloniki (reference number: 297/14-05-2018). A purposive sampling method was used to reach possible participants. Interviews were conducted to explore the views of nineteen (*N* = 19) participants. To enhance the diversity of the sample, participants were recruited using a multimodal recruitment technique. Participants were recruited from the oncology department of a large university teaching hospital in Thessaloniki (‘Papageorgiou’ Hospital), interviewers’ (A-MT) personal acquaintances, and referrals made by physicians with different specialties who are involved in the care of young women with cancer or their FP (oncologists, breast surgeons and fertility health care professionals). Furthermore, researchers used the snowball sampling technique to recruit participants using a small pool of initial participants as informants. Possible participants were contacted face-to-face to be given information and then confirm participation and find a suitable time and place for carrying out phenomenological interviews.

The interview guide was developed prior to conducting interviews and reviewed by a bioethicist with experience in reproductive ethics (PV) and a qualitative researcher (A-MT). Semistructured questions were developed on the basis of the results of a literature review on the topic of interest. It was slightly refined after the initial results of a few interviews to make the interview guide more probing. Questions mostly focused on women’s understanding of biological motherhood and FP, how they were given information on this, how they felt about doing it, and what were the influences behind the decisions of participants considering FP. The interview guide used in this study was developed for this study. The questions included in it are presented in Supplementary file 1.

Data collection ceased only when data saturation was reached. Field notes were taken immediately after each interview and were taken into account by researchers when conducting data analysis. Validity was observed by using maximum variance in participant selection. Reflexive thinking was employed throughout the research process to reduce unintentional personal bias and enhance the trustworthiness of the study. The participants did not provide feedback on the findings.

The interviews were conducted at interviewees’ preferred times in quiet and neutral places of their choice with only the interviewer (A-MT) and participant present. The interviews were audio-recorded and then transcribed verbatim. After carefully reading and rereading each interview transcript, the researchers coded units that were similar in meaning. Codes with similar meanings were grouped into subcategories. Then, subcategories were condensed into categories, which in turn were grouped into themes. Disagreements among the authors were addressed through discussion. The research was conducted by a multidisciplinary research panel including bioethicists, lawyers, oncologists and obstetrician-gynaecologists specializing in human reproduction.

## Results

The number of subjects interviewed was nineteen (*N* = 19). The participant characteristics are presented analytically in Table 1.

**Table 1:** Participant characteristics

Participant	Age Ranges	Type of Cancer	Children	Level of Education	Marital status
P1	26–35	Breast Cancer	0	Tertiary	Marriage-like relationship
P2	36–45	Cervical Cancer	0	Tertiary	Single
P3	26–35	Breast Cancer	0	Tertiary	Single
P4	36–45	Breast Cancer	2	Secondary	Married
P5	26–35	Breast Cancer	1	Tertiary	Married
P6	36–45	Breast Cancer	1	Tertiary	Married
P7	26–35	Breast Cancer	1	Tertiary	Married
P8	36–45	Breast Cancer	1	Secondary	Married
P9	26–35	Cervical Cancer	0	Secondary	Single
P10	26–35	Breast Cancer	0	Tertiary	Marriage-like relationship
P11	26–35	Breast Cancer	0	Tertiary	Married
P12	26–35	Colon Cancer	0	Tertiary	Married
P13	36–45	Breast Cancer	2	Secondary	Married
P14	36–45	Breast Cancer	0	Tertiary	Single
P15	18–25	Ovarian Cancer	0	Tertiary	Single
P16	36–45	Stomach Cancer	0	Secondary	Marriage-like relationship
P17	26–35	Breast Cancer	0	Tertiary	Single
P18	36–45	Lymphoma	0	Tertiary	Single
P19	36–45	Breast Cancer	0	Tertiary	Single

The thematic data analysis revealed five major themes and eight subthemes (Table 2).

**Table 2:** Major themes and subthemes

Theme	Subtheme
2. Many patients had a strong and deeply held desire for biological offspring.	1.1. Facial resemblance and similarities of offspring to mothers and a good relationship with spouses/partners emerged as reasons behind the desire for biological offspring. 1.2. Cancer diagnosis can weaken the women’s desire to reproduce. 1.3. Cancer diagnosis can clarify and strengthen the women’s desire to reproduce.
2. Patients preferred oocyte cryopreservation to other fertility preservation (FP) options.	2.1. Unwillingness to preserve embryos by cryopreservation for different reasons. 2.2. Surrogacy for FP emerged as the ultimum refugium option.
3. The provided information was unclear and deficient.	3.1. Lack of clear information. 3.2. Deficient information. 3.3. Satisfactory information was only provided after patient questions.
4. Patients decided for themselves. They made decisions together with their husbands and partners for some specific reasons.	
5. Religious, social and financial reasons did not emerge as factors that affect participants’ FP decisions.	

### Many patients had a strong and deeply held desire for biological offspring

#### Facial resemblance and similarities of offspring to mothers and a good relationship with spouses/partners emerged as reasons behind the desire for biological offspring

Data analysis concluded that, with few exceptions, most participants had a strong deeply held desire for biological offspring. Participants said that their desire for biological offspring reflected a woman’s desire to create a human being, that is, a continuation of herself, with similar in traits and appearance.

P9 said,

…having biological offspring is very important. It is like continuing yourself. It is fascinating to know what your child will look like, whether or not they look like you, what traits of yours they will have.

In a similar vein, P1 said,

I find it charming to see someone like myself grow up, mainly as evolution of myself ….

Furthermore, participants said that their desire for biological offspring depends on the existence of an appropriate partner. P2 said,

… to me, becoming a parent is directly connected to companionship. The right partner and chemistry within the couple at the time of becoming parents are very important. I have always wanted someone to be coresponsible….

In a similar vein were Participants P3 and P19. Their interview quotes are presented in Supplementary file 2.

One participant said that she desires to have biological offspring because it is *‘something very beautiful’* (P6), with another participant saying,

‘*I need to experience pregnancy.’* (P2).

Remarkably, none of the participants considered having a biological child as the only solution.

#### Cancer diagnosis can weaken women’s desire to reproduce

Some participants stated that it was not very important for them to become mothers. However, while they had clearly expressed their previous desire to have biological offspring, they reported a range of cancer diagnosis-related reasons weakening their original desire for biological offspring. Perhaps their original desire was not strong enough. One reason was that these participants were not nulliparous before the cancer diagnosis. P13 (46 years old, who already had two children at the time of diagnosis and was interested in having a third child) said that if she had not had a child already, having been diagnosed with cancer, she would think of preserving fertility even if she had to postpone cancer treatment. However, if chemotherapy had to start *“so immediately”*, then her priority would be fighting the disease. The participant’s voice emphasized the term *“so immediately”.* In a similar vein were other participants who already had a child at the time of diagnosis. Furthermore, among the cancer diagnosis-related reasons reported (in many cases cumulatively) as weakening their original desire for biological offspring were woman’s quality of life that might have been negatively impacted in case of distressing recurrent failure in assisted reproduction (the P1, P10) or from negative consequences of FP methods (P2) and at any rate from the oncological disease itself. A mother’s low quality of life negatively impacts the quality of life of the future offspring. An ill mother would be unable to meet her parental duties (P9, the P1, P12, P5, P6). For instance, Participant 6 said,

*‘I would not take the risk of leaving a child without his or her mother, wittingly, while I know my disease.’* (P6).

Furthermore, participants expressed their fear that cancer itself could be passed down from parents to children (the P6, P10, P19). Other reasons weakening participants’ desire to reproduce were the patient’s disorientation from fighting her disease (P2) and lack of a partner. Participant 2 said, *‘I have always wanted someone to be coresponsible…’* Moreover, participants emphasized the patient’s advanced reproductive age, given that in all likelihood she would undergo assisted reproduction many years later due to anticancer treatment (P2). Finally, financial reasons were among the reasons weakening the desire to preserve fertility, which were always reported cumulatively with other reasons (P14, P2, P18, P7, P11).

Finally, it is to be added that some participants said they felt compromised by the idea of childlessness and pursued FP given the threat of cancer (P15, P16, P18).

#### Cancer diagnosis can clarify and strengthen women’s desire to reproduce

Cancer diagnosis may act as two sides of the same coin. It not only can weaken the desire for biological offspring but can also make it stronger.

Another category of participants included those with a very strong (deeply held) original desire to reproduce who made it clear at the beginning of the interview that they would proceed with FP even if their life was threatened. Note, however, that later in the course of the interview, they said they would only forgo FP in case of extremely high risk for their life or a child’s well-being.

Participant 12 said,

When I was informed about [possible infertility and] fertility preservation, I cried a lot. Note, however, that when I heard about the cancer diagnosis I didn’t!

The participant would rather give priority to fertility in case of disease-fertility conflict. She said it was very important for her to bear a child, although the cancer she suffered was aggressive.

Interestingly, Participant 11 stressed,

*‘After cancer diagnosis, the first thing I thought of was my fertility rather than if I shall live or not”* and that *“the procedure of preserving fertility was not carried out in a good mood and this bothered me psychologically more than the very procedure of fighting cancer.’* However, she pointed out that both the challenge she was experiencing while fighting cancer and her respect for the moral status of the human embryo, which might eventually be destroyed because it might be redundant, were important obstacles to preserving fertility.

Furthermore, cancer diagnosis can clarify the internal attitude of FCPs towards having biological offspring because it brings them face-to-face with the dilemma of preserving fertility or not, a condition/question in which the patient has to come to a decision immediately. Although Participant 3 did not want to have children before her diagnosis, she said,

*‘…lack of the possibility to choose changed my mind*… *I would like to have the option.’* Participant 2 was in a similar vein. While Participant 1 was previously at a loss to make a reproductive choice, after cancer diagnosis, she turned out to be clearly positive towards having a child.

### Patients preferred oocyte cryopreservation to other fertility preservation (FP) options

Some participants were reluctant to opt for embryo cryopreservation due to religious or nonreligious reasons. Furthermore, almost all participants expressed more or less strong concerns about surrogacy as an FP option for various reasons.

#### Unwillingness to preserve embryos by cryopreservation for different reasons

Some participants (P9, P11, P16, P17) clearly expressed their unwillingness to opt for embryo cryopreservation because they were reluctant to choose this option out of respect for the moral status of the early human embryo for religious or other reasons or because they had received inadequate information about the particular FP method. Participants’ interview quotes are presented in Supplementary file 2.

Participant 19 said she would not opt for embryo cryopreservation, not for moral reasons but because she was of the view that having offspring is strictly related to the existence of a partnership. She did not know if they would be together in the future. She said,

I did not proceed to embryo cryopreservation not because I am morally committed, but because I do not know what the relationship status with my partner will be after I have gone through all this [the disease] … I would not like to be committed to something that would affect future decisions regarding having offspring.

Among participants in this study, respect for the human embryo’s moral status is discussed as a barrier to FP and has emerged as a major barrier to opting for embryo cryopreservation.

#### Surrogacy for FP emerged as an *ultimum refugium* option

All participants expressed more or less strong concerns about the use of surrogacy as an FP option for various reasons.

Participants said that they could resort to a surrogate uterus only with persons closely related to them. Otherwise, moral precautions arise since the surrogate uterus exploits a foreign female body, and trust matters. P11 said,

Surrogacy would be one of my last choices… I cannot pay a woman to give me a part of her body.

Participant 12 said that surrogacy is a method that

I would not opt for, because it hurts the woman that gives birth, even if the offspring is not hers [genetically].

However, the participant has had discussions with her sister to become a surrogate for her [the participant]. Participant 13 said that she would not enter into the process of surrogacy because *‘as a human, it does not seem to be ethically correct to me…’* Participant 9 said that surrogacy is something that looks unfamiliar to her. However, she said that she might opt for it under certain circumstances, for instance, if the surrogate mother is *‘a woman in a very close relationship to me, mother or other relative… this could make me opt for it [surrogacy].’*

Participant 13 said,

Surrogate motherhood makes me feel strange; nevertheless, if I could not have the option to have offspring, I would try to find the proper woman to get pregnant for me, maybe a woman with whom I have an intimate relationship, like a sister, mother, a person who is very close to me….

Other participants considered surrogacy *‘to be the ultimate refugium’* (P14 P17, P18). Two participants expressed a strong negative attitude towards opting for surrogacy as an FP method. Note, however, that these participants already had children and did not feel a strong desire for having other biological offspring (P5, P6).

### The provided information was unclear and deficient

#### Lack of clear information

This emerged as a highly recurrent finding within the data analysis. Participants reported that the information they were given about their FP was not clear. A diffusion of responsibility for providing information about FP was identified among oncologists, surgeons and fertility specialists, who sometimes had different opinions. The following quotation is representative to illustrate this point.

Participant 19 said,

As to [the side effects of] ovarian stimulation during the procedure of fertility preservation, I did not receive a clear answer. This made me feel involved in a precarious situation because I would not like to do something that could harm my health. Furthermore, I did not know how harmful it could be, how to evaluate my priorities.

Participants P1, P9 and P11 were in a similar vein. Their interview quotes are presented in Supplementary file 2.

While different medical specialties are involved in the field of oncofertility, the vagueness of provided information may be due to physicians’ unwillingness (irrespective of their specialty) to take full responsibility for the information provided to a patient.

While the oncologist allowed Participant 14 to proceed with FP,

…at that time, he realized for the first time that he had not mentioned the subject of fertility preservation at all, he justified himself saying that he believed information was given to me by the surgeon. The surgeon considered that I was informed by the oncologist and, in this way, I had never been informed for the possibility to opt for fertility preservation.

#### Deficient information

Α good number of participants said that the information they received was deficient and that they were not satisfied with the process within which information was provided to them. This emerged from the data analysis as a recurrent finding. The following quotations are representative of this point.

Participant 2 said that she was not satisfied with the information she had received, and she noticed,

Ι consider that if the excellent grade is 10, I would give this process a 3.5. I wish the medical specialists’ group had provided me with an in-depth and overall view [of what they had to inform me about], from the time of diagnosis….

Participants P11 and P14 were in a similar vein. Their interview quotes are presented in Supplementary file 2.

#### Satisfactory information was only provided after patient questions

Many participants considered that they were given adequate information and declared that they were satisfied with it. However, they noted that the information they were given (perceived as adequate) was received after having asked physicians to provide them with further information about specific aspects of FP options. The following quotation is representative of this point.

Participant 19 said,

…my physicians ‘informed’ me, in general. However, I am satisfied with the information I finally got. I received answers to most of my questions because I had asked these questions. I do not know if I would have been given adequate information if I had been less ‘pressing’….

Other participants were satisfied with the information they received because they wittingly avoided asking further questions of the physicians (though they could) for various reasons. Participants preferred to trust their physicians and avoid taking responsibility for FP decisions. This was a highly recurrent finding. While Participant 3 declared she felt satisfied with the information given, she said she had not asked questions because she thought she knew all she needed to know. She was certain that hormone therapy would negatively influence her cancer. Her top priority was to overcome her oncological disease as soon as possible. In a similar vein, Participant 4 declared satisfaction from the information she was given, but she admitted she had not asked much because she already had and did not need to learn more.

Participants P5, P6, P7, P12, P13, P15 and P16 were in a similar vein. Their interview quotes are presented in Supplementary file 2.

Four participants (P1, P14, P17 and P8) declared that they were not satisfied with the information they received. Note, however, that two of them (P14 and P8) stressed that they had avoided asking physicians questions for various reasons.

While Participant 17 was reluctant to receive further information, she considered the given information very deficient in the area of FP. The participant said,

As a patient, I did not wish for much information because I felt overwhelmed so that I could not function. I said no, I do not want to know so much [information]….

Furthermore, the participant complained *‘…no physician informed me about the risk of cancer activation by hormones provided within the fertility preservation process…’*.

Furthermore, Participant 8 did not ask questions of the physicians because she did not trust them enough.

Finally, in some cases, physicians may wittingly avoid providing FP information to patients due to their belief that there is no point to do this given that the chemotherapy could not be postponed (P18).

From data analysis, it emerged that physicians may avoid providing information about the available FP options for the following reasons: (a) The patient is not at an advanced reproductive age, and the possibility of having future offspring is very high (P15 18 years old, P1 30 years old). (b) Physicians must hurry to initiate anticancer therapy as soon as possible, and there is no time to lose (P18). (c) The patient already has one child (P13, P4). (d) The type of cancer is hormone-sensitive cancer, and the available FP method involves the administration of hormones. Participants P1, P2, and P3 said that their physicians said they would not suggest FP. In the same vein, Participant 9 said, *‘My physicians informed me that I had betteravoid proceeding with fertility preservation.’* In a similar vein, Participant 11 said, *‘… my surgeon had a negative attitude towards proceeding with fertility preservation before surgery.’* (e) The patient is not interested in proceeding with FP (P3, P6, P2). For instance, Participant 2 said,

*‘Although my physician advised me to make a referral to a fertility specialist, I decided not to do this…While I had an appointment with a fertility specialist, I have never been there…’*.

### Patients decided for themselves. They made decisions together with their husbands and partners for specific reasons

Most patients had finally decided for themselves if they would apply for preservation of fertility methods. Husbands’/partners’ opinions were simply taken into account. The same holds for their family or friends. However, if husbands/partners contributed (or would be contributing) their own genetic material, as in the case of embryo cryopreservation, their opinion was seriously taken into consideration in women’s FP decision-making process. Two participants pointed out that the husband/partner plays a pivotal role in preserving fertility if he has offered his own genetic material, as in the case of embryo cryopreservation. Participant 9 said,

The partner plays a very important role if the child that will be born also has his genetic material. However, since fertility preservation is an invasive procedure regarding the woman’s body, finally I would make a decision on my own concerning how to proceed, bearing in mind his opinion.

In the same vein was Participant 10. Additionally, financial reasons might make the husband/partner play a decisive role. Participant 4 said that the decision to proceed to FP is not a decision that she could make alone but jointly with her husband/partner since the cost of FP is great.

Participants gave the impression that their trust in an intimate person plays a significant role in decision-making. Participant 6 was a pharmacist and said she trusted her husband/partner’s opinion because he was also a pharmacist.

For almost all participants, parents did not play a crucial role, with the exception of Participant 9, who trusted them because they *‘know her well’.* The parents of Participant 15 (a very young woman) decided jointly with the physician, whom they absolutely trusted. She also trusted her physician too much. She said, *‘if he had something to tell me, he would have said it to me.’*

### Religious, social and financial reasons did not emerge as factors that affect participants’ FP decisions

All participants except for two (P16 and P9) did not report religious barriers to proceeding with FP. Importantly, the same holds true for participants who described themselves as religious or spiritual. P16 said, *‘I believe that this is not a matter that my spiritual leader [confessor] can solve.’* Financial factors were always taken into consideration for decision-making, without being a determinant factor affecting the participants’ final decision. Some participants mentioned financial factors only in addition to other reasons supporting their decision not to apply for FP (P2, P7, P11). None of the participants reported financial factors as an exclusive reason for not proceeding with FP. Participants always referred to financial factors (highlighting them to a greater or lesser extent) in addition to other reasons. Finally, none of the participants considered social reasons as factors of particular significance.

## Discussion

While most of the participants in this study suffered from breast cancer, six out of nineteen participants suffered from cancer primarily located in other organs: cervical (two participants), ovarian, stomach, colon, and lymphoma. It should be noted that ‘the incidence of colorectal cancer among premenopausal women is increasing’ [17]. The diversity of cancer types in our small sample is in line with the available literature. Importantly, despite the enormous need of premenopausal women with cancer for FP prior to treatment, only a small percentage of these patients actually managed to do so.

Participants in this study experienced a lack of close collaboration among all relevant stakeholders involved in their FP decisions. That situation goes against the promotion of the patient’s autonomy and well-being. As oncofertility is an emerging and multidisciplinary field [3], the produced international or national guidelines should be multidisciplinary [18, 19]. FP guidelines have been implemented since 2013. In 2020, the European Society of Human Reproduction and Embryology (ESHRE) published a detailed guideline ‘written by a multidisciplinary group with gynaecologists and fertility specialists, oncologists, a psychologist, a bioethicist, an embryologist, a scientist, and patient representatives’ [18]. The same goes for other guidelines developed in the US, Spain and France [19–22].

Furthermore, comprehensive fertility counselling and optimal care should be provided by a multidisciplinary team of health providers, including ‘oncologists, reproductive endocrinologists, mental health counsellors and clinical researchers’ [23]. A close and strong collaborative effort of all relevant stakeholders is required [3, 18, 19, 23–26]. The appropriate FP method in a given case must follow multidisciplinary strategies. It must be carefully selected upon shared decision-making [18, 27, 28]. The selection of the most appropriate option should be individualized and may be determined by factors such as patient age, patient characteristics, desire for conception, disease, treatment plan and socioeconomic status [19, 25]. FP decision-making in women with cancer is a complex process [29].

Many participants in this study felt that they had received inadequate information. However, some participants felt that they had been adequately informed on their own initiative. These participants were classified into two categories: those who declined further information and those who sought more information and asked further questions of health providers. That is, health providers would only give enough information on the patient’s request.

It is essential that health care professionals (especially oncologists and haematologists) provide adequate information to reproductively active women with cancer about the feasibility of preserving fertility as early as possible prior to the initiation of anticancer treatment [18–20]. The American Society of Clinical Oncology (ASCO) [20] and the European Society of Human Reproduction and Embryology (ESHRE) [18] embrace this view. Furthermore, many authors share the consideration that FP in cancer patients protects their mental health and promotes their quality of life, enables patients to better cope with their cancer-related stress, can ‘boost their confidence in treatment’, ‘reduces their long-term regret or disappointment concerning fertility’, and facilitates patients in making well-informed decisions about their cancer care [3, 19, 21, 22, 26, 29, 30]. It is argued that it is physicians’ moral obligation [3, 18, 20, 21]. The information provided should be tailored to the needs of the various subgroups of women [31]. Importantly, the provision of information should be combined with ‘appropriate and effective fertility-related’ psychological support (fertility counselling) [26]. Nevertheless, oncofertility counselling is ‘underutilized’ for female patients for various reasons [3, 2, 24, 28, 32–34].

Indeed, only a small percentage of young women with cancer receive suboptimal counselling and/or receive referrals to FP services. This emerged as a recurrent finding in the literature review [2, 3, 9, 24, 35]. A retrospective study has shown that ‘of all the 918 surveyed cancer survivors who had potential reproductively toxic cancer treatments, 61% of them were counselled by an oncologist about their infertility risk, but only 5% of them visited a fertility specialist and 4% of them ultimately chose to preserve their fertility’ [3]. Covelli et al. cited a literature review to suggest that despite the existing guidelines (i.e., ASCO guidelines, ‘an estimated 50% of women with cancer remain uninformed about the potential for cancer-related infertility, and even fewer are referred to fertility specialists’) [36]. This happens for a variety of reasons [24]. For example, it is argued in the literature that while ‘66–100% of patients with cancer expressed a need for fertility information’, ‘about half of patients (43–62%) felt that relevant information was provided inadequately and that their information needs were not addressed’ [35]. Suboptimal counselling is a factor that serves as a critical barrier to FP [2]. Furthermore, it should be noted that the volume and content of FP information that should be provided to reproductively active women with cancer are not clear and commonly accepted. Importantly, the ESHRE provided detailed guidelines for addressing this issue [18].

Most participants in this study had not received a referral for FP options. Although women with cancer may be focused initially on their diagnosis, to increase the likelihood of future child-bearing potential, reproductively active women with a cancer diagnosis should be promptly referred to reproductive specialists before treatment initiation [2, 21, 28, 29, 37].

In this study, data analysis implicates physicians’ lack of knowledge about cancer-related FP. The literature states that a lack of information for patients and, ‘unfortunately’, a lack of knowledge for professionals are critical barriers to FP services [9]. The findings of a study conducted by Covelli et al. suggest that medical education has not kept pace with FP technologies, which has left many clinicians uninformed about them” [36]. Health providers should be prepared to discuss FP options with their female patients who receive a cancer diagnosis, provide adequate information to them, assist them in making the best possible choices and refer them properly as soon as possible [21, 24, 33].

All participants in this study were offered narrow FP options, mostly oocyte and embryo cryopreservation. Selection of the appropriate FP option for a particular patient includes a variety of factors cited in a study conducted by Logan and Anazodo [26].

In this study, physicians remained concerned about the safety of controlled ovarian stimulation (COS) for FP before initiating anticancer treatment, particularly in patients with hormone-sensitive cancer. It is true that the safety of FP treatments in cancer patients is a matter of paramount importance. However, this seems to be due to their lack of experience in communicating state-of-the-art knowledge. While FP provided before starting cancer treatment can significantly delay cancer treatment initiation, it is argued that performing FP treatments involving COS before anticancer treatment in young women with breast cancer does not seem to be associated with increased cancer recurrence or mortality [10, 38–40].

Oocyte and embryo cryopreservation are widely available, long- and well-established preservation options that are most effective for reproductively active women with cancer [3, 18, 20, 21, 29, 41]. The literature states that ‘embryo cryopreservation has slightly higher success than oocyte cryopreservation in achieving pregnancy’ [42]. Embryo cryopreservation is considered ‘the most widely available option’; however, it requires the existence of a partner or the woman’s openness to sperm donation [24, 29]. Given that cryopreserved (frozen) embryos could be considered the joint property of the couple, this method of FP can give rise to difficult questions such as who gets the embryos in case of relationship breakdown [18, 29].

Almost all participants in this study were not given information about the technique of OTC as an FP option for selected patients. OTC, in vitro oocyte maturation, ovarian transposition, ovarian suppression, and adjuvant therapy are included among the experimental FP options for these patients [3, 18]. OTC, which has substantially expanded the field of FP, seems to be the front-runner among the experimental options and is on the verge of becoming a well-established FP option [30, 41, 42]. Importantly, ovarian tissue can restore ovarian function and does not require prior ovarian stimulation [21, 42]. While OTC remains an experimental FP option, it can be an option for specific patients [17, 18, 21, 42]. Currently, OTC is indicated in patients whose fertility is at very high risk due to anticancer treatment [22]. For instance, ESHRE acknowledged that OTC can be performed when there is not sufficient time for COS [18]. OTC is considered ‘a secure tool in human fertility preservation’ [43]. In Sweden, OTC is an option available at many reproductive health care centres [39].

Moreover, the literature states that ‘in vitro oocyte maturation (IVM) can also be considered, and in some cases, there may be a possibility of combining different approaches’ [18].

While embryo cryopreservation is a method for FP that gives rise to ethical concerns and legal questions, oocyte and embryo cryopreservation are currently routinely applied methods for FP in young women with cancer. However, some young women with cancer cannot undergo routinely applied FP methods for medical reasons. These women might undergo other less routine FP methods. OTC is currently the most common technique for FP in (selected) cancer patients. While OTC seems to be currently the front-runner among the less routine FP methods, IVM is also a similar method. Importantly, OTC as an FP method is currently an ongoing process. The standardization of protocols for OTC and OTT is currently ongoing. At any rate, it should be highlighted that there are discrepancies between countries regarding the accessibility of FP services to patients. These discrepancies arise because of different ethical considerations. A joint effort to achieve resonance of counselling in the field of oncofertility is required [44].

Cancer diagnosis enhanced the desire for biological offspring in participants in this study (especially in participants with a strong desire for children at the time of diagnosis). However, in some participants (especially those with a weak previous desire for children), cancer diagnosis reduced their desire for biological offspring for various reasons reported by them. An unfulfilled desire for biological offspring can be associated with impaired mental health [26]. Thus, it is not surprising that young women’s desire for biological offspring seems to remain strong after cancer diagnosis or even after cancer treatment, especially in patients who are nulliparous at the time of diagnosis [27]. This is in line with findings in this study. Follow-up studies have shown that women who have a desire for biological parenthood at the time of initial cancer treatment are more prone to seek and receive FP consultations [24]. Surprisingly, it is argued that ‘it would be wrong to assume cancer patients with advanced disease…have no desire to preserve their fertility’ [24].

Furthermore, in this study, financial issues emerged as a fairly important factor affecting FP decisions. Patients with cancer should be made aware of the available financial assistance programs to become more flexible in addressing ‘this complex and heterogeneous landscape’ [21] ‘during an uncertain and challenging time in their lives’ [33]. Financial constraints are critical obstacles that prevent young breast cancer and other cancer patients from accessing FP services [33, 45]. Assisted reproduction services are too expensive in many countries, and many women with cancer have no access to FP services not only in low- or middle-income countries but also in high-income countries [24]. It is argued that in the US, ‘utilization of financial assistance for FP was low despite literature pointing to the need for such assistance’ [46]. It is argued that in the US, better insurance coverage could facilitate access to FP services and ultimately ‘improve long-term cancer survivorship’ [47]. The use of fertility services may increase financial hardship among cancer patients in countries where lack of or inadequate insurance coverage prevents cancer patients from accessing FP services [24]. Nevertheless, there are countries where these services are totally or partly subsidized. In Sweden, FP in cancer patients is publicly funded [39]. In the Czech Republic, FP in cancer patients is partially covered by health insurance companies [9].

In this study, religious belief emerged from data analysis as a slightly important or not at all important factor affecting FP decisions. The different religions vary considerably in their attitudes and beliefs on the morality of artificial reproduction. ‘While most forms of artificial reproduction are acceptable in Hinduism and Buddhism, its acceptance in Christianity and Islam is variable depending on the branches or sects within the religious group’ [24]. For instance, all forms of assisted reproductive techniques are unacceptable in Roman Catholicism, while acceptance is varied among Orthodox Christians [24].

Religious beliefs are included among the factors influencing the patient’s decision on which preservation options may be available to them [26]. Furthermore, in line with the results of this study, it is argued that women with cancer may have religious or ethical objections to embryo cryopreservation [21].

The barriers to FP utilization in young women with cancer are multifactorial, including patient factors, health care provider-related factors, socioeconomic factors and institutional factors [24]. Ojo et al. cited further details [24].

Ultimately and most importantly, it should be noted that as the distress of making a fertility decision is further complicated by the concurrent distress of the cancer diagnosis, patients are most likely to become easily ‘overwhelmed and ill-equipped to manage this complex multistep decision-making process’ [26]. Other studies are in a similar vein [33, 48, 49]. Kim et al. conducted a survey completed by 204 participants. They found that 64% of participants ‘reported that they were too overwhelmed at the time of their cancer diagnosis to consider FP options’ [48]. Logan et al. conducted a systematic literature review and found that some women with cancer endorsed the need for information at the time of diagnosis, while other women highlighted ‘the importance of receiving fertility information during cancer treatment decision-making and in follow-up’ [49]. At any rate, for women with a cancer diagnosis, choosing FP is a complex emotional process of making ‘one of the most difficult decisions ever made’ [26].

### Implications for future policies

As assisted reproduction is further developing in Greece, relevant services are becoming accessible for larger parts of the Greek population. However, at present, there are no official data regarding patients who are undergoing FP procedures prior to cancer treatments. The results of this research can contribute to identifying the needs of patients together with gaps in health services and assist further improvements in the management of premenopausal women diagnosed with cancer.

### Strengths and limitations of the study

Most participants were recruited through the snowball sampling technique. This enhances the diversity of the study and can be regarded as a strength. Furthermore, the sample consisted of women with various types of cancer. This can also be seen as a strength. However, this study should be interpreted in light of certain limitations. Almost all participants were between 30 and 45 years old, with the exception of only one participant who was a very young woman (eighteen years old). Moreover, potential self-selection bias cannot be ruled out. Women who were particularly interested in preserving their fertility were more likely to have responded to our call for research participation. In addition, recall bias cannot be excluded to some extent, at least with regard to certain findings. Finally, participants were not asked for feedback or to check the consistency between their intentions and the results obtained by the researchers. This fact limits the reliability of the study in terms of confirmability.

## Conclusions

A variety of distinct themes and subthemes emerged from the analysis of the interview data. The cancer diagnosis emerged as a factor that considerably affects the women’s attitudes towards biological parenthood: It can further increase their (strong) previous desire or decrease their previous (weak) desire. Women with a recent cancer diagnosis had not received adequate and multidisciplinary counselling, including clear and adequate information. However, participants felt satisfied with the information they received because they received the information they wanted, either by asking questions or by being in denial about the need to be informed (i.e., because they felt overwhelmed after the cancer diagnosis). Embryo cryopreservation emerged as a less desirable FP option for women with cancer. Participants showed respect for human embryos, not always for religious reasons. Surrogacy emerged as the last resort option for most participants. Religious, social and financial factors played a secondary (if any) role in women’s decision-making about FP. Finally, male partners’ opinions played a secondary role in most participants’ decision-making about FP. If embryo cryopreservation was the selected option, partners would have a say because they were contributing their genetic material. The findings that emerged from the data analysis were partly consistent with prior literature. However, we identified some interesting nuances that are of clinical importance. The results of this study may serve as a starting point for future research.

### Electronic supplementary material

Below is the link to the electronic supplementary material.

Supplementary Material 1

Supplementary Material 2

## Data Availability

The transcripts of the full interviews that were collected and qualitatively analysed in the current study are not available for reasons of confidentiality. The redacted transcripts that were used and analysed as part of the current study can be made available by the corresponding author upon reasonable request.

## Abbreviations

COS: Controlled Ovarian Stimulation
IVM: In vitro oocyte Maturation
EFS: Event-free Survival
FP: Fertility Preservation
ART: Assisted Reproductive Technologies
ASCO: American Society of Clinical Oncology
ESHRE: European Society of Human Reproduction and Embryology
ASRM: American Society for Reproductive Medicine
HCP: Health Care Professionals
Q-o-L: Quality of Life
