# Supplementary material for: Perceptions and experiences of fertility preservation in female patients with cancer in Greece
Source: BMC Womens Health. 2024 Feb 9;24:108. doi: 10.1186/s12905-024-02955-x (PMC10858603; doi:10.1186/s12905-024-02955-x)
Supplement: Supplementary file 1 — Supplementary Material 1 [file 12905_2024_2955_MOESM1_ESM.docx]

**Online Supplement**

**Supplement 1: The interview guide used in this study**

1. What does it mean for you to be a child’s biological mother? (*a grand tour question to make the participant comfortable*).
2. What is your understanding of fertility preservation as a means of overcoming the risk of cancer treatment-induced infertility?
3. How did you experience the process of considering fertility preservation as a means of overcoming the risk of cancer treatment-induced infertility?
4. What were the factors that influenced your decision-making when considering fertility preservation?
5. How satisfied were you with the information you were given on this topic, and why?
6. Did you find discussing fertility preservation with physicians or other health professionals to be easy?

As the questionnaires were semistructured, they included questions such as ‘When you were diagnosed with cancer, had you reached your desired family size [had as many children as you hoped to have]?’ and ‘After diagnosis, but before starting treatment, did you see a fertility specialist to talk about fertility preservation?’.
