# Supplementary material for: Perceptions and experiences of fertility preservation in female patients with cancer in Greece
Source: BMC Womens Health. 2024 Feb 9;24:108. doi: 10.1186/s12905-024-02955-x (PMC10858603; doi:10.1186/s12905-024-02955-x)
Supplement: Supplementary file 2 — Supplementary Material 2 [file 12905_2024_2955_MOESM2_ESM.docx]

**Online Supplement**

**Supplement 2: Participants’ interview quotes**

**Major themes and subthemes**

**Many patients had a strong and deeply held desire for biological offspring.**

**Facial resemblance and similarities of offspring to mothers and a good relationship with spouses/partners emerged as reasons behind the desire for biological offspring.**

Furthermore, Participant P3 said,

*“After [cancer] diagnosis, I would possibly think about an offspring only in relation to a partner”.*

In a similar vein, Participant P19 said,

*“…having a child results from the bond between yourself to your partner”.*

**Cancer diagnosis can weaken women’s desire to reproduce.**

**Cancer diagnosis can clarify and strengthen women’s desire to reproduce.**

**Patients preferred oocyte cryopreservation to other fertility preservation (FP) options.**

**Unwillingness to preserve embryos by cryopreservation for different reasons.**

Furthermore, Participant 16 declared her reluctance to opt for embryo cryopreservation for religious reasons. She said,

*“I believe in God and feel that the fertilized oocyte is an early existence of man…Oocyte is something that goes to waste every month. It is not the same as a fertilized oocyte”.*

She noted that she would not want to throw away any embryo; for this reason, she would make as many pregnancy attempts as the number of fertilized oocytes.

Participant 9 said that she would not proceed with the cryopreservation of embryos *“for religious reasons”* without providing any further detail.

Participant 11 was a hundred percent against the cryopreservation of embryos because of her increased respect for early embryo moral status, which was not related to religiosity or spirituality. She said,

*“I feel that I create embryos, some of which will be used (to achieve pregnancy), with the other embryos being thrown away. I do not accept it for moral reasons.”*

Participant 17 would not proceed with embryo cryopreservation not because of moral reasons but due to the lack of adequate information she had received. She said,

*“I think I would not do it. It is something unfamiliar to me… not because of ethical concerns… Maybe I have not yet received needed information …”.*

**Surrogacy for FP emerged as an *ultimum refugium* option.**

**The provided information was unclear and deficient.**

**Lack of clear information.**

Participant 11 reported “*controversial views”* between physicians (oncologist, surgeon and fertility specialist).

Participant 9 said,

*“I wish I had received more clear information…. I felt that there were questions regarding many aspects of fertility preservation, which were not clearly answered by physicians.”* She added,

*“I want [the physician] to admit that a clear and accurate answer on this subject is lacking yet… I need a well-informed physician to become a well-informed patient*.*”*

While Participant 1 declared she felt satisfied with the information she was given, she added,

*“…fertility preservation is a grey zone … they neither said to me it is permitted nor it is prohibited… in clarity…”*

**Deficient information.**

Furthermore, Participant 14 said that no physician informed her about FP, although she had no children and wanted to have one. She contacted a fertility gynaecologist, and the procedure of preserving fertility was immediately on the way, without being further informed.

Similarly, Participant 11 said,

*“…the way the fertility physician communicated with me was not a decent one. It was cold, absolute, pressing. He made me anxious and said I had to give an answer ‘at the soonest possible time’… He did not give me even a single day to think.”*

**Satisfactory information was only provided after patient questions.**

Furthermore, Participant 5 was satisfied with the information she received from her physicians. She said,

*“I could have asked for more information…[however] because of the “shock” Ι experienced when I was informed about diagnosis, I could not ask for more …”*

Moreover, Participant 6 said,

*“…the information I received from physicians was ok. It encompassed all I needed to know…”*

In the same vein, Participant 12 learned about the possibility of preserving fertility from a person who was not a physician! Finally, however, she felt satisfied with the information she received and said,

*“The information provided encompassed all I needed to know…I did not want to know anything else. Ι felt satisfied. My only complaint was that the communication should be more polite.”*

Similarly, Participant 13 said,

*“I learned what I had to learn”.*

P15 agreed with this statement.

Participant 16 said,

*“I wanted to be offered a treatment, which I had no choice but to accept …I would have liked not to have to make a [difficult] decision. I wanted to feel that I could trust my physician…”*

In a similar vein, Participant 7 said that she trusted her physician. She said,

*“I did not ask much.”*

**Patients decided for themselves. They made decisions together with their husbands and partners for specific reasons.**

**Religious, social and financial reasons did not emerge as factors that affected participants’ fertility preservation decisions.**
